# Dissolving Hyaluronic Acid Filler: The Diffusion Barrier to Hyaluronidase Efficacy

**DOI:** 10.1111/jocd.70818

**Published:** 2026-03-25

**Authors:** G. Murray, S. Khoshnaw, P. Velthuis

**Affiliations:** ^1^ Kings College London Institute of Pharmaceutical Science London UK; ^2^ Erasmus Medical Center Rotterdam GD the Netherlands

**Keywords:** aesthetic complications, complication management, cross‐linked hyaluronic acid, dermal fillers, enzymatic degradation, Fick's law of diffusion, hyaluronic acid, hyaluronidase, ultrasound‐guided injection

To the Editor,

A Guardian article noted that some women resort to surgical facelifts after fillers fail to dissolve properly [1]. Since hyaluronidase does not always work as intended, alternative methods are being explored. For instance, Wang et al. reported repeatedly using needling to induce pinpoint bleeding to facilitate filler removal after unsuccessful treatment of a vascular event [2]. With a better understanding of the physiochemistry, such measures may be unnecessary.

Hyaluronidase is the enzyme of choice for dissolving unwanted hyaluronic acid (HA) filler. It cleaves the β‐1,4‐glycosidic bonds of hyaluronic acid (HA), reducing cross‐linked gel polymers into smaller fragments that can be cleared physiologically. However, clinical experience and experimental data [3] both show that hyaluronidase often fails to achieve complete dissolution when injected without imaging or mixing. The enzyme does not freely diffuse through the tissue to dissolve any nearby HA filler.

This limitation can be explained by Fick's law of diffusion, which describes how solute flux (J) across a medium depends on the diffusion coefficient (D) and the concentration gradient. Here, D refers to the ability of hyaluronidase to diffuse within the HA matrix. The dense polymer network and small pore size of cross‐linked HA fillers reduce this coefficient by restricting macromolecular movement [4], such as hyaluronidase. Experimental studies using time‐lapse video microscopy have shown that most cross‐linked HA fillers can be degraded by hyaluronidase in a dose‐ and time‐dependent manner [5]. The degree of cross‐linking had a greater impact on degradability than the HA concentration, with higher cross‐link density resulting in slower degradation. Differences in hyaluronidase formulation and labeled potency, including ovine‐derived, bovine‐derived, and recombinant human preparations available in a wide range of dosages, may also influence the effective enzyme activity in vivo. Additionally, in vivo HA filler deposits may be compartmentalized within adipose or connective tissue or become encapsulated over time, increasing the diffusion distance (dx) and creating additional physical barriers to enzyme access. Incomplete mixing further compounds this problem. When hyaluronidase and HA filler remain as distinct physical phases (one being a fluid, the other a gel), the interface available for diffusion is limited, thereby reducing enzymatic flux, and this may result in incomplete degradation. Combined with the short half‐life of hyaluronidase in tissue, these factors limit the likelihood of full enzymatic contact with the entire gel mass. Consequently, diffusion alone is insufficient to ensure enzymatic contact with the entire gel mass.

These diffusion constraints underscore the importance of direct and targeted delivery of hyaluronidase. Ultrasound‐guided injections enable visualization of filler deposits and ensure accurate enzyme placement within the HA filler, increasing local enzyme concentration, contact surface, and improving efficacy. Gentle postinjection manipulation may further enhance interfacial mixing and accelerate degradation.

In summary, the limited diffusion of hyaluronidase within cross‐linked HA fillers, governed by a low diffusion coefficient, minimal contact surface area, and tissue compartmentalization, explains the frequent failure of blind injections. Effective filler dissolution depends on overcoming these physical diffusion barriers through precise enzyme placement, appropriate dosing, and, where possible, ultrasound guidance (Table 1).

**TABLE 1:** Strategies to overcome barriers to the clinical efficacy of hyaluronidase (enzyme) in hyaluronic acid filler.

Barriers	Strategy
Filler located in the tissue compartment, separate from the enzyme injection	Use ultrasound guidance to place the enzyme exactly in the tissue compartment where the filler is located
Limited interface between the enzyme and the filler substance	Inject different areas inside the filler pocket
	Deliver the enzyme with a firm, targeted injection
	Gently manipulate the area postinjection to improve contact
Slow diffusion in dense polymer‐network in HA filler	Increase enzyme concentration or dosage
Highly cross‐linked filler degrades slowly	Repeat enzyme injections

## Funding

The authors have nothing to report.

## Consent

The authors have nothing to report.

## Conflicts of Interest

The authors declare no conflicts of interest.

## Data Availability

Data sharing not applicable to this article as no datasets were generated or analysed during the current study.
